# Supplementary material for: Foliar salt spray exclusion and tissue tolerance underlie local adaptation to oceanic salt spray
Source: Am J Bot. 2026 Aug 17;113(8):e70251. doi: 10.1002/ajb2.70251 (PMC13496090; doi:10.1002/ajb2.70251)
Supplement: Supplementary file 3 [file AJB2-113-e70251-s003.docx]

**Appendix S11.** Comparison of abaxial stomatal traits between ecotypes. Points indicate LSMs for each accession, with 15 individuals measured per accession, and error bars show confidence intervals. *β*- and *P*-values indicate the effect and significance of inland ecotype on the trait from general linear mixed models. (A) Stomatal density in stomata against ecotype. (B) Stomatal length against ecotype. (C) Estimated fraction of epidermis area allocated to stomata against ecotype.
