# Supplementary material for: Foliar salt spray exclusion and tissue tolerance underlie local adaptation to oceanic salt spray
Source: Am J Bot. 2026 Aug 17;113(8):e70251. doi: 10.1002/ajb2.70251 (PMC13496090; doi:10.1002/ajb2.70251)
Supplement: Supplementary file 4 [file AJB2-113-e70251-s004.docx]

**Appendix S1.** (A) Coastal perennial ecotype in coastal habitat (OSW, Oswald West State Park, Oregon, USA, 45.761083°N, 123.966567°W). (B) Inland annual ecotype in inland habitat (MOR, Alder Creek Ranch on Morelli Rd, Occidental, California, USA, 38.4297°N, 122.951617°W).
