# Supplementary material for: Foliar salt spray exclusion and tissue tolerance underlie local adaptation to oceanic salt spray
Source: Am J Bot. 2026 Aug 17;113(8):e70251. doi: 10.1002/ajb2.70251 (PMC13496090; doi:10.1002/ajb2.70251)
Supplement: Supplementary file 5 [file AJB2-113-e70251-s005.docx]

**Appendix S6.** Mean sodium and water levels in leaves of coastal and inland accessions 20 h after treatment with deionized water or salt spray. Four leaves were analyzed for each accession in each treatment. Error bars show standard errors of the mean. Shapes indicate accession, as described in the legend for Figure 2. (A) Molarity of sodium in leaves for each ecotype following spray treatment, assuming all Na is in solution in the volume of water in the leaf at the time of sampling. (B) Amount of Na per unit leaf area for each ecotype and treatment. (C) Succulence as (fresh mass – dry mass) / leaf area for each ecotype and treatment.
