# Supplementary material for: Foliar salt spray exclusion and tissue tolerance underlie local adaptation to oceanic salt spray
Source: Am J Bot. 2026 Aug 17;113(8):e70251. doi: 10.1002/ajb2.70251 (PMC13496090; doi:10.1002/ajb2.70251)
Supplement: Supplementary file 7 [file AJB2-113-e70251-s007.docx]

**Appendix S9.** Wettability traits in coastal and inland accessions, with means from 15 analyzed leaves for each accession. Error bars show standard errors of the mean. Shapes indicate accession as described in the legend for Figure 2. (A, B) Contact angle of a drop of water placed on the (A) adaxial surface and (B) abaxial surface of the leaf. (C) Result of leaf water drop adhesion assay.
