# Supplementary material for: Foliar salt spray exclusion and tissue tolerance underlie local adaptation to oceanic salt spray
Source: Am J Bot. 2026 Aug 17;113(8):e70251. doi: 10.1002/ajb2.70251 (PMC13496090; doi:10.1002/ajb2.70251)
Supplement: Supplementary file 8 [file AJB2-113-e70251-s008.docx]

**Appendix S12.** Stomatal traits (*y*-axis) compared between coastal and inland accessions. Points indicate means for each accession; error bars show standard errors of the mean. Traits were measured for 15 individuals from each accession for all traits except *g*_sw_, where six leaves from 2–3 individuals were measured for each accession. (A) Adaxial stomatal density. (B) Adaxial stomatal length. (C) Estimated fraction of adaxial epidermal area allocated to stomata, (D) Abaxial stomatal density. (E) Abaxial stomatal length. (F) Estimated fraction of abaxial epidermal area allocated to stomata. (G) Stomatal conductance of adaxial surface. (H) Amphistomy as adaxial stomatal count/(abaxial stomatal count + adaxial stomatal count), where dashed line at amphistomy = 0.5 corresponds to equal numbers of stomata on both leaf sides.
