# Supplementary material for: Foliar salt spray exclusion and tissue tolerance underlie local adaptation to oceanic salt spray
Source: Am J Bot. 2026 Aug 17;113(8):e70251. doi: 10.1002/ajb2.70251 (PMC13496090; doi:10.1002/ajb2.70251)
Supplement: Supplementary file 11 [file AJB2-113-e70251-s011.docx]

**Appendix S7.** Mean leaf mass per area in coastal and inland accessions 20 h after salt spray or control treatment. Four leaves were analyzed for each accession in each treatment. Error bars show standard errors of the mean. Shapes indicate accession as described in the legend for Figure 2.
