# Supplementary material for: Foliar salt spray exclusion and tissue tolerance underlie local adaptation to oceanic salt spray
Source: Am J Bot. 2026 Aug 17;113(8):e70251. doi: 10.1002/ajb2.70251 (PMC13496090; doi:10.1002/ajb2.70251)
Supplement: Supplementary file 17 [file AJB2-113-e70251-s014.docx]

**Appendix S3.** Standard curve for sodium quantification by MP-AES analysis.
